# Active module identification in intracellular networks using a memetic algorithm with a new binary decoding scheme

**DOI:** 10.1186/s12864-017-3495-y

**Published:** 2017-03-14

**Authors:** Dong Li, Zhisong Pan, Guyu Hu, Zexuan Zhu, Shan He

**Affiliations:** 1 School of Computer Science, University of Birmingham, Birmingham, B15 2TT UK; 2 PLA University of Science and Technology, Nanjing, 210007 China; 3 College of Computer Science and Software Engineering, Shenzhen University, Shenzhen, 518060 China

**Keywords:** Memetic algorithm, Module identification, Connectedness, Module size

## Abstract

**Background:**

Active modules are connected regions in biological network which show significant changes in expression over particular conditions. The identification of such modules is important since it may reveal the regulatory and signaling mechanisms that associate with a given cellular response.

**Results:**

In this paper, we propose a novel active module identification algorithm based on a memetic algorithm. We propose a novel encoding/decoding scheme to ensure the connectedness of the identified active modules. Based on the scheme, we also design and incorporate a local search operator into the memetic algorithm to improve its performance.

**Conclusion:**

The effectiveness of proposed algorithm is validated on both small and large protein interaction networks.

## Background

With the increased use of high-throughput experimental data such as gene expression profiles, protein-protein interactions and metabolic response [1], we are able to gain better understanding of the molecular mechanisms of biological functions. Because molecules interact with each other to exert biological functions, it is important to understand not only the activity of individual molecules, but also their interaction. In the past decade, network biology approaches which explicitly model the molecule interactions as graphs or complex networks have been intensively used. One of the primary tasks is to explore topological properties of biological networks, such as community structure [2] and network motifs [3]. Though the topology of a biological network does not always precisely reflects the function or even disease-determined regions [4], they may have some overlapped components, which then can be related back to biological functions.

Active module identification is one of the most important network biology analysis algorithm, which is able to reveal the regulatory and signaling mechanisms of a given cellular response [5]. The algorithm aims to find an connected regions over certain biological networks that show significant changes under certain conditions. In the seminal work of [5], the authors first constructed protein-protein interaction network where the nodes represent proteins, and edges represent the physical interactions between a pair of proteins. Node scores which indicate the significance of expression changes over certain conditions were calculated from the gene expression data and then assigned to the nodes. The active module identification problem was formulated as a combination optimization problem, which aims to search a subnetwork that maximize the aggregated score.

This combinatorial optimization problem turns out to be NP-hard [5], which is equivalent to finding a maximum weight clique in a weighted graph, a famous NP-complete problem [6]. As effective tools to solve combinatorial problems, metaheuristic algorithms have been widely applied to search satisfied solutions [7, 8]. The original paper [5] proposed to use simulated annealing (SA), a generic probabilistic metaheuristic to solve this problem. Other methods include extended simulated annealing [9], greedy algorithm [10, 11], graph-based heuristic algorithm [12] and genetic algorithm (GA) [13, 14]. A comprehensive review of this filed can be found in [15].

Binary encoding is the most common solution representation for active module identification using metaheuristic optimization algorithms such as SA or GA. In this encoding, the module in *n*-nodes network can be represented by membership vector **x**∈{0,1}^*n*^, where *x*_*i*_=1 means *i*-node belongs to the module. One of the prerequisites to use this representation is to ensure the connectedness of the solution, which is not only a biological requirement for resulting subgraphs (connected subgraph means reachable interactions inside the module). Without the connectedness constraint, the maximal objective may corresponds to a set of unrelated top-ranked nodes. Unfortunately most related works mentioned above either did not consider this non-trivial constraints, or did not tackle this aspect efficiently.

Another problem of using generic metaheuristic optimization algorithms is that the search operators, i.e., perturbation [5], mutation and crossover [14], are not specifically designed for active module identification, which might result in mediocre search performance in terms of speed and accuracy. In our previous works, we have shown that by incorporating local search operators into generic metaheuristic optimization algorithms, we can significantly improve the speed and accuracy for community detection in large scale biological networks [16, 17].

In this paper, in order to address the connectedness problem, we first propose an effective encoding/decoding scheme. Based on the representation, we propose a local search operator and then embed it into a memetic framework. We have evaluated the proposed method for both simulated and real-world data, which shows the superior performance over other algorithms.

## Methods

### Active module identification

Commonly the an interaction network is represented as an undirected graph *G*=(*V*,*E*), nodes in *V* represent genes, and edges in *E* represent the interactions between two genes. We can assign each gene *i* a *p*-value *p*_*i*_ to indicate the significance of expression changes over certain conditions. Then we can obtain a *z*-score *z*_*i*_=*Φ* ^−1^(1−*p*_*i*_) for each gene, where *Φ* ^−1^ is the inverse of normal CDF.

To find a subnetwork which has high nodes scores, the aggregation *z*-score of subnetwork *A* *z*_*A*_ is defined as [5]:

1 $$ z_{A}=\frac{1}{\sqrt{k}}\sum_{i\in A}z_{i},  $$

where *k* is the number of genes in *A*. In order to get subnetwork which has higher aggregation *z*-score compared with a random set of genes, it is suggested to use a corrected subnet score *s*_*A*_ [5]:

2 $$  s_{A}=\frac{z_{A}-\mu_{k}}{\sigma_{k}},  $$

where the mean *μ*_*k*_ and standard deviation *σ*_*k*_ are computed based on a Monte Carlo approach, taking several rounds of randomly sampling *k* genes from the network. The simplified problem of finding highest score module in an undirected network, which consider the subnetwork score is the sum of each node’s score, is formally defined as following:

#### **Problem 1**

Given a graph *G*=(*V*,*E*) with vertex weight **z**= [ *z*_*v*_] for each *v*∈*V*, find a connected subnetworks *S*=(*V*_*S*_,*E*_*S*_) of G with maximal weight $f(S) = \sum _{v\in V_{S}}z_{v}$.

In order to solve Problem 1, which is a NP-hard combinatorial optimization problem, meta-heuristics algorithms have been applied. For example, simulated annealing was used in [5]. In each iteration, if toggling the state of a randomly picked node can increase *s*_*A*_ of expected subnetworks, then one choose to toggle it; otherwise to toggle it with certain probability. After a number of iterations, a set of high score subnetworks can be obtained. In [14], based on binary encoding scheme, Genetic Algorithm with genetic operators such as mutation and crossover has been proposed to search for active modules.

### New binary encoding/decoding scheme for active module identification

Despite the biological insightful results obtained from the algorithms mentioned above, one important detail was omitted in the papers: how to ensure the connectedness of the resulting subgraph after applying heuristic operators such as toggling, mutation or crossover. This detail is important because without ensuring the connectedness of a candidate solution, the identification of active modules could be trivial, i.e., a set of isolated top-ranked nodes.

In the source code provided by the original authors (jActiveModules, a plug-in for Cytoscape [18]), the authors employed a sophisticated way to check whether toggling one node of a membership vector is feasible, i.e., whether the toggling will affect the connectedness of the candidate solution, which makes the whole algorithm slow. Specifically, given a candidate solution, i.e., a subset of nodes, an additional HashMap has to be maintained to stores the pairwise elements {*n* *o* *d* *e*,*c* *o* *m* *p*}, which indicates each node and its component (connected subnetwork), respectively, during the whole progress. After toggling, the algorithm will check this HashMap to see whether the operator affects the connectedness of resulted subnetworks. Such operations leads to both running time and memory overhead.

In this paper, we propose a simple but fast binary encoding/decoding scheme, which does not require the HashMap nor explicit operations when add or remove current nodes. Our binary encoding scheme is the same as used in [14], i.e., a binary vector of *n* binary values of which each represents the membership of the node (*x*_*i*_=1 means *i*-node belongs to the module). The key difference is the decoding scheme. Wile the previous work [14] did not consider the connectedness constraint. Specifically, we conduct the connected components finding (CCF) algorithm on the binary vector presented subset, and then extract the connected subnetworks. Decoding scheme based on CCF algorithm as described in Algorithm 1, where Breadth-first search (BFS) is used to recursively to find the node’s neighbors.



Since there are multiple connected subgraphs in a candidate solution, the fitness calculation can be flexible. In the simplest case, we can use the subgraph with the highest aggregated node score. However, no matter how we calculate the fitness function, genetic meta-heuristics algorithms can be directly applied based on the encoding/decoding scheme. For example, if we use SA, in each iteration, we decide to add or remove a randomly picked node by the same criterion: if toggling the state of the selected node *c* can increase *s*_*A*_ of the subnetwork *A* with the highest aggregated node score, then we choose to toggle it; otherwise to toggle it with certain probability *p*. Compared with original mechanism of jActiveModules in Cytoscape, this decoding is computational tractable and easy to implement.

The connected components finding Algorithm 1 is actually based on breadth-first search (BFS) on a (sub)graph, requiring time complexity *O*(|*V* ^′^|+|*E* ^′^|) where |*V* ^′^| and |*E* ^′^| are the number of nodes and edges of the current set respectively. Notice that this time complexity is only equivalent to one case to toggle a node in jActiveModules in theory.

### Memetic algorithm

Evolutionary algorithm (EA) is a powerful global optimization to solve combinatorial optimization problems. Inspired by biological evolution, a typical EA uses operators such as selection, crossover and mutation to improve the candidate solutions [19]. Parameters for an EA are number of iterations *T*, population size *P*, crossover probability *p*_*c*_ and mutation probability *p*_*m*_.

Memetic algorithm (MA) improved standard EA by enabling individuals to perform local refinements [20]. Numerous effective local search (LS) methods have been developed and incorporated into MA to obtain state-of-the-art results in various applications [21–23]. A recent review of MA can be found in [24]. Algorithm 2 describes a common framework of MA, where the standard mutation operation is replaced by a local search operator. Being similar to conventional GA algorithms which partially prevent the “local optimum” problem by mutation and crossover mechanisms, Algorithm 2 uses an enhanced mutation step. With enough number of evolutionary generations, this algorithm is supposed to convergence.



According our encoding/decoding scheme, each candidate solution consists of several connected subgraphs, we define the highest score of these subgraphs as the fitness of **x**, denoted by *F*(**x**). For multiple modules identification, we use a module extraction mechanism, i.e. to identify one active module each time and then extract it from the background network, which is left for next round.

For the local search part, here we mainly consider a simple greedy search strategy. We pick all individuals in the population with probability *p*_*LS*_ and conduct *M* times of toggle on current individual where *M*<*N*. Finally we replace each chosen individual with the best scored one, followed by other genetic operators. More operations as in [22] to conduct local search could be applied here.

It is necessary to make sure the identified module has reasonable **size** when toggling nodes. Both extreme small and large module can make the interpretation difficult. But the objective (2) itself cannot prevent large modules. Neither original work [5] nor GA based method [14] proposed mechanisms to achieve reasonable sized modules. Furthermore, to maximize objective (2) may lead to single gene module or very large component in practice. As long as one large module (e.g. containing 1,000 genes) is connected and has high aggregated score, then this module may be found using general algorithm 2.

Here we make a simple modification to the mutation operator in GA and local search operator in MA to constrain the module size to be desired: as long as the number of candidate genes (number of ‘1’s in encoding vector) exceeds some threshold *N*_*max*_, there will be no more potential nodes added to the subset. On the contrary, if the module size is going to be smaller than predefined threshold *N*_*min*_, there will be no more potential nodes removed out from the current subset.

The procedure of local search is described as in Algorithm 3. The whole procedure of MA for active module identification is combining general memetic framework 2 and the local search strategy. For evolutionary operations in the whole procedure, we chose the commonly used one-point crossover.



The computational complexity for memetic Algorithm 2 is *O*(*T* *P*) without local refinements. The expected computational complexity of whole algorithm with greedy search is thus *O*(*T* *P*+*T* *M*(|*V* ^′^|+|*E* ^′^|)) where |*V* ^′^| and |*E* ^′^| are the number of nodes and edges of a candidate solution subgraph respectively. If we consider almost half of the whole nodes may get involved in evolution and normally the number of edges |*E* ^′^| in subgraph approximately at the same level of the number of nodes |*V* ^′^|, the simplified complexity of the whole algorithm should be *O*(*T* *P*+*T* *M* *N*). Generally the size of population *P* is small compared with the network size *N*, which makes the latter dominate the running time. And the number of local search trails *M* in each inner iteration also has an impact on the efficiency. In theory the sophisticated mechanism of jActiveModule can also be used here, but it would makes the fitness evaluation more difficult. And the space requirement is higher due to the HashMap.

## Results and discussion

### Module connectedness validation

**Fig. 1:**
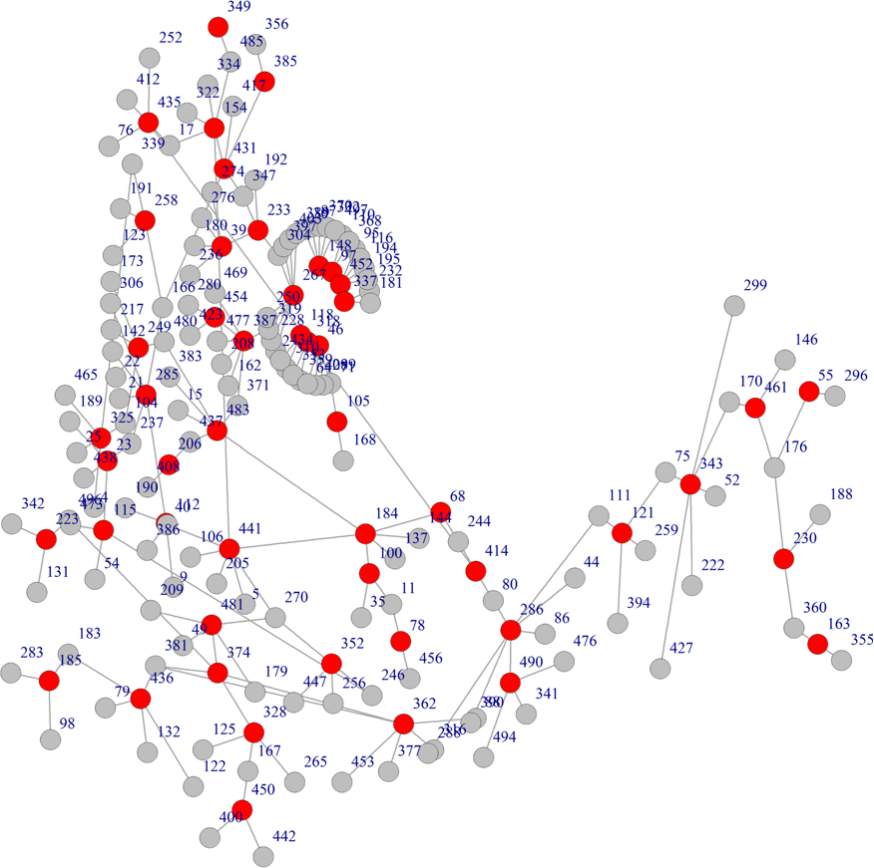
Modules identified by GA on simulated data. The red nodes are not connected though they are supposed to be

**Fig. 2:**
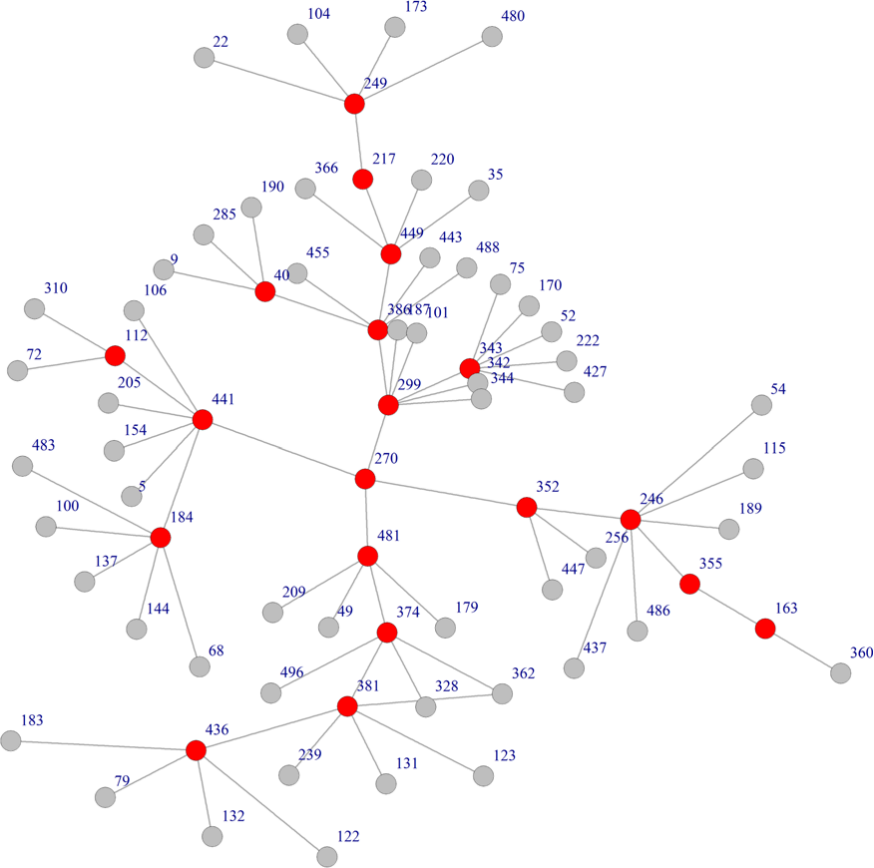
Modules identified by modified GA with proposed encoding scheme on the same simulated data as in Fig. 1. The red nodes are connected

First of all, we validate if the modules identified by proposed algorithm are connected. The baseline algorithm is a simple GA with basic binary encoding scheme without connectedness guarantee to search highly scored module in molecular networks. We use a simulated interaction network with 500 nodes and 1000 edges, to just validate the connectedness property. Figure 1 showed the resulted module, and the red nodes are in subset of resulted module and gray ones are their neighbors but not included in the subset. We can see that the original subset is not connected at nodes like 185, 400 and 163 etc, which are isolated from large set of red nodes. If we use the same GA algorithm with the proposed encoding mechanism in section 3, we can get a different result as Fig. 2 shows. With the same input and algorithmic parameters, the red nodes are now connected in the identified active module. The standard GA (modified from COSINE [14]) and visualization code are available at https://github.com/fairmiracle/EAModules.

### Yeast PPI network

We first validate the proposed algorithm on a small real protein-protein network with 329 proteins in Yeast [25]. The *p*-values on each nodes show the significance of gene expression changes in response to a single perturbation: a strain with a complete deletion of the GAL80 gene versus wild type yeast. The network structure data and expression values are available from Cytoscape sample data. The constructed network has 329 nodes and 358 edges. And the goal is to find a top-scoring subnetwork which show significant response to the perturbation.

We compare the performance of three algorithms using the encoding method in section 3: simulated annealing (SA), genetic algorithm (GA) and the proposed memetic algorithm (MA). In order to compare SA with other two EAs fairly, we run SA *P* (also the population size in GA and MA) times and select the best result, since SA is viewed as a single population GA. The number of iterations *T* for all algorithm is 10000, and temperatures decrease from 1.0 to 0.01 for SA. Other evolutionary parameters are crossover rate *p*_*c*_=0.9 for GA and MA, mutation rate *p*_*m*_=0.9 for GA and local search iterations *M*=10 for MA. In GA and MA we also reserve the best individual in each iteration for stability. We run each algorithm 50 times with randomly initialization and then compare the performance w.r.t highest module score and corresponding module size.

**Fig. 3:**
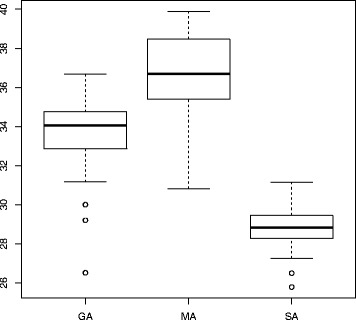
Statistical comparison of performance w.r.t module scores from three algorithms: genetic algorithm, memetic algorithm and simulated annealing

Figure 3 summarizes the results in terms of module score based on 50 trails. We can see MA can achieve slightly higher mean score than GA, and both are better than SA. One-Way Analysis of Variance (ANOVA) is used to determine differences between results from three algorithms, with *p*−*v* *a* *l* *u* *e*<2.2*e* ^−16^. And a paired sample t-test is used to tell the difference between GA and MA, with *p*−*v* *a* *l* *u* *e*<1.19*e* ^−5^.

**Fig. 4:**
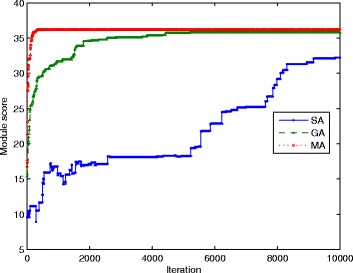
Convergence rate comparison of three algorithms: genetic algorithm, memetic algorithm and simulated annealing from one trail. MA is the first to reaches the stable status

Besides the quality of module, we also compare the rate of convergence of three algorithms, to see how objective improves along with iterations. We define the best objective value in population as the indicator in each iteration. According to Fig. 4, MA reaches the stable objective earlier than GA. The local search scheme could make sure the performance of MA is no worse than basic GA, and the monotonic selection leads to early convergence compared with GA, at the cost of longer running time of local search. Both GA and MA get higher objective than SA, which needs much more iterations to reach high score.

### Human PPI network

In order to check the biological relevance of identified modules by proposed algorithm, we apply it on the real world protein-protein interactions (PPI) network. The background PPI network for *homo sapiens* is obtained from two updated databases: BioGRID [26] Release 3.4.138 and STRING v10.0 [27], specifically 9606.protein.links.v10.txt. The BioGRID for *homo sapiens* has 362,775 interactions while STRING stores 8,548,002 protein pairs, with a combined score ranging from 150 to 999 for each link. The gene expression profile comes from GEO35103 controlled by the differentiation of Th17 cell, which is considered to play a key role in pathogenesis of autoimmune and inflammatory diseases [28]. The expression profile contains 48,000 probes (genes), and 28,870 were kept after the following process: 1) remove probes those do not have gene symbols; 2) remove probes with more than 20% of missing values or NAs; 3) replace the rest missing data with mean value of the row they belong to. Further we select 5003 significantly expressed genes from all of them using limma [29]. The gene filtering algorithm selects some potentially important candidates and reduce network size. Finally we select PPI pairs according to match of expression probes.

For BioGRID we simply match the gene names for each probe of expression profile. But STRING uses the protein name (start with ENSP), thus we need to match that with official symbols (like ARF5) with database Ensembl Genes 84 [30], and select the corresponded genes. The source code for genes selection and construction procedure of PPI network from multiple data sources is available at https://github.com/fairmiracle/PPINet.

The network constructed from BioGRID has 2327 nodes and STRING has 1602 nodes, with 1480 nodes in common. We conduct the algorithm 2 on both networks, and use a module extraction method to identify multiple modules from this network, i.e. to identify one active module each time and then extract it from the background network, which is left for next round. The largest size of each module is 100. The full gene symbols lists of modules are provided in supplementary materials (at https://github.com/fairmiracle/EAModules/tree/master/examples/Supplementary, where “GSE35103FromString_MA.txt” means the modules identified from STRING based PPI network using MA algorithm, and each module is stored as plain text by module score, gene ids and official gene symbols). We can also see that under the same condition, MA could achieve higher scored modules than GA.

In order to validate the identified modules, we follow the gene set enrichment analysis [31] and use various updated tools, including basic gene ontology (GO) database (http://geneontology.org) and Analysis in STRING, integrative and interactive web-based tools like GeneMANIA (http://genemania.org) [32]. The basic idea of annotating a given gene list is to compare it with known knowledge database. The *P*-value is calculated by the following formula (3).

3 $$  P=\sum\limits_{x=1}^{n}\frac{{M\choose x}{N-M\choose n-x}}{{N\choose n}}.  $$

**Fig. 5:**
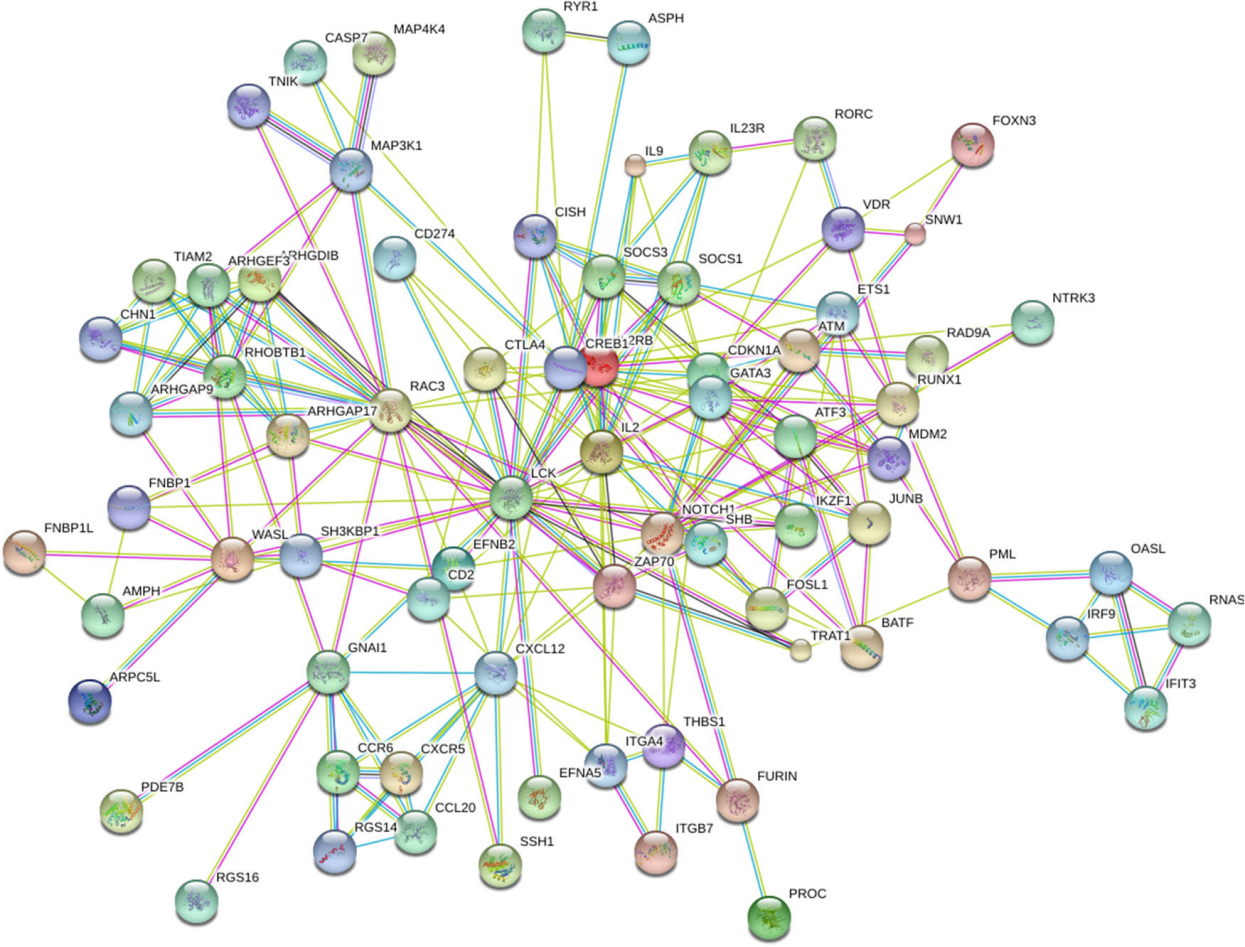
The first identified module plotted by STRING, where edges represent both known interactions including curated databases and experimentally determined and predicted interactions such as gene neighborhood and gene co-occurrence

Generally speaking, larger module tends to be enriched multiple biological functions, which may not be very relevant to each other. The first module identified from STRING PPI network contains 76 genes and according to GeneMANIA [32], among all potential links inside the module, there are 51.63% co-expression links, 33.59% are physical interactions and 4.16% are pathways. The top biological processes and pathways related to this module are listed in Table 1. We can see several general responses found by STRING, and the hub nodes in this module shown as in Fig. 5 also indicate general important genes related to receptor signaling and signal transduction (also see http://bit.ly/2a87HTB). While functions given by GeneMANIA show that these functions are intensively involved in Th17 cell differentiation. Several items are also claimed in a recent publication [33], which is consistent with the experimental settings.

**Table 1:** Enrichment analysis result of the first module

Biological process (GO) given by STRING			
Pathway ID	Pathway description	Count	FDR
GO.0007166	Cell surface receptor signaling pathway	39	1.85E-17
GO.0007165	Signal transduction	52	1.35E-16
GO.0044700	Single organism signaling	51	1.11E-14
GO.0007154	Cell communication	51	2.36E-14
GO.0051716	Cellular response to stimulus	54	5.15E-14
KEGG pathway given by STRING			
5166	HTLV-I infection	10	6.45E-06
4630	Jak-STAT signaling pathway	8	1.22E-05
4380	Osteoclast differentiation	7	2.95E-05
5202	Transcriptional misregulation in cancer	7	0.000154
04151	PI3K-Akt signaling pathway	9	0.000194
Functions given by GeneMANIA			
Index	Function	FDR	Coverage
1	T cell differentiation	5.63e-12	13/90
2	lymphocyte differentiation	5.63e-12	15/144
3	leukocyte differentiation	6.95e-12	17/226
4	Positive regulation of leukocyte activation	1.87e-11	15/166
5	Positive regulation of cell activation	2.55e-11	15/172
6	Regulation of leukocyte activation	1.01e-10	16/232
7	T cell activation	1.57e-10	16/241

**Fig. 6:**
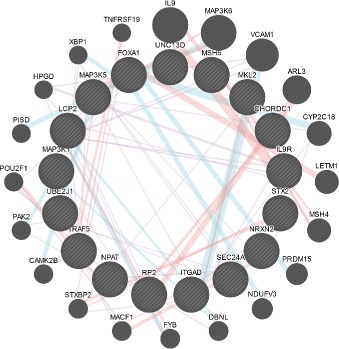
The relatively small module plotted by GeneMANIA, where most of edges are co-expression links according to previous studies

The smaller module tends to play more specific roles in the process. Figure 6 plotted by GeneMANIA [32] shows the interactions between these 17 genes, and 87.84% of them are co-expression links according to previous studies. The function is more about pathways, like Fc-epsilon receptor signaling and Fc receptor signaling. Related genes contained in this module are MAP3K1, MAP3K5 and MAP3K6, mitogen-activated protein kinase kinase, which play central roles in the regulation of cell survival and differentiation. The connection between MAP3k and Th17 differentiation is supported by [34], through encoding MEKK1 which controls both B and T cell proliferation. And MEKK1 regulates Cdkn1b expression in Th17 cells. Other processes enriched by the module are also mentioned in a recent study [35].

Different sources of protein-protein interactions also make an impact. From the comparison between modules between BioGRID and STRING networks, we can see that they share some functions such as Fc-epsilon receptor signaling pathway, but they are not totally the same. Interactions in BioGRID are largely rely on high-throughput datasets and previous studies, which makes the identified module less focused to some functions. Irreverent supporting materials make the set of genes has lower coverage and higher FDR, given by functional enrichment report by GeneMANIA. In contrast, STRING has many experimental and predicted interactions [27], and the combined score of links can further help to pick more reliable edges of PPI network. Identified modules from this network tend to have more significant biological meanings. Take the first module (http://bit.ly/2asI0Nw) for example, gene ontology tells the hierarchical biological process of this module by starting with regulation of tyrosine phosphorylation of Stat3 protein. The Stat3 has been shown to be a master regulator of Th17 cell differentiation [36] and related immune pathways.

## Conclusion

Searching for connected subnetworks in biological networks is essentially a combinatorial optimization problem, which can be solved by various metaheuristic methods. We design a direct strategy on a set of node to get connected subnetworks, thus avoid complicated graph divide operations. And the binary encoding can be used in general heuristic optimization algorithms like simulated annealing and genetic algorithm. And the GA is further improved by a memetic algorithmic framework embedded with local search operators. Empirical studies on real networks shows the effectiveness and efficiency of this strategy.

Future works can be considered in two different aspects. From the network model, how to derived effective algorithmic model to deal with directed and weighted network is of interests. The PPI network itself is weighted and confidence score of interactions may affect results. And the direction of some edges has biological meanings as well. From the evolutionary algorithm view, the method used in this paper is rather superficial and various state-of-the-art techniques have not been employed. Further improvements on EA may make it more efficient in handling large-scale networks.

## References

[1] Wang Y, Eddy JA, Price ND (2012). Reconstruction of genome-scale metabolic models for 126 human tissues using mcadre. BMC Syst Biol.

[2] Girvan M, Newman ME (2002). Community structure in social and biological networks. Proc Natl Acad Sci.

[3] Milo R, Shen-Orr S, Itzkovitz S, Kashtan N, Chklovskii D, Alon U (2002). Network motifs: simple building blocks of complex networks. Science.

[4] Barabási AL, Gulbahce N, Loscalzo J (2011). Network medicine: a network-based approach to human disease. Nat Rev Genet.

[5] Ideker T, Ozier O, Schwikowski B, Siegel AF (2002). Discovering regulatory and signalling circuits in molecular interaction networks. Bioinformatics.

[6] Karp RM (1972). Reducibility among combinatorial problems. Complexity of Computer Computations.

[7] Huang Q, White T, Jia G, Musolesi M, Turan N, Tang K, He S, Heath JK, Yao X (2012). Community detection using cooperative co-evolutionary differential evolution. International Conference on Parallel Problem Solving from Nature.

[8] Jia G, Cai Z, Musolesi M, Wang Y, Tennant DA, Weber RJ, Heath JK, He S (2012). Community detection in social and biological networks using differential evolution. Learning and Intelligent Optimization.

[9] Guo Z, Li Y, Gong X, Yao C, Ma W, Wang D, Li Y, Zhu J, Zhang M, Yang D (2007). Edge-based scoring and searching method for identifying condition-responsive protein–protein interaction sub-network. Bioinformatics.

[10] Ulitsky I, Shamir R (2007). Identification of functional modules using network topology and high-throughput data. BMC Syst Biol.

[11] Ulitsky I, Shamir R (2009). Identifying functional modules using expression profiles and confidence-scored protein interactions. Bioinformatics.

[12] Rajagopalan D, Agarwal P (2005). Inferring pathways from gene lists using a literature-derived network of biological relationships. Bioinformatics.

[13] Klammer M, Godl K, Tebbe A, Schaab C (2010). Identifying differentially regulated subnetworks from phosphoproteomic data. BMC Bioinforma.

[14] Ma H, Schadt EE, Kaplan LM, Zhao H (2011). Cosine: Condition-specific sub-network identification using a global optimization method. Bioinformatics.

[15] Mitra K, Carvunis AR, Ramesh SK, Ideker T (2013). Integrative approaches for finding modular structure in biological networks. Nat Rev Genet.

[16] Liu Y, Tennant DA, Zhu Z, Heath JK, Yao X, He S (2014). Dime: a scalable disease module identification algorithm with application to glioma progression. PloS ONE.

[17] He S, Zhu Z, Jia G, Tennant D, Huang Q, Tang K, Heath J, Musolesi M, Yao X (2016). Cooperative co-evolutionary module identification with application to cancer disease module discovery. IEEE Trans Evol Comput.

[18] Shannon P, Markiel A, Ozier O, Baliga NS, Wang JT, Ramage D, Amin N, Schwikowski B, Ideker T (2003). Cytoscape: a software environment for integrated models of biomolecular interaction networks. Genome Res.

[19] Golberg DE (1989). Genetic algorithms in search, optimization, and machine learning. Addion Wesley.

[20] Moscato P (1989). On evolution, search, optimization, genetic algorithms and martial arts: Towards memetic algorithms. Caltech Concurr Comput program C3P Rep.

[21] Ishibuchi H, Yoshida T, Murata T (2003). Balance between genetic search and local search in memetic algorithms for multiobjective permutation flowshop scheduling. IEEE Trans Evol Comput.

[22] Zhu Z, Ong YS, Dash M (2007). Wrapper–filter feature selection algorithm using a memetic framework. IEEE Trans Syst Man Cybern B Cybern.

[23] Tang K, Mei Y, Yao X (2009). Memetic algorithm with extended neighborhood search for capacitated arc routing problems. IEEE Trans Evol Comput.

[24] Neri F, Cotta C (2012). Memetic algorithms and memetic computing optimization: A literature review. Swarm Evol Comput.

[25] Ideker T, Thorsson V, Ranish JA, Christmas R, Buhler J, Eng JK, Bumgarner R, Goodlett DR, Aebersold R, Hood L (2001). Integrated genomic and proteomic analyses of a systematically perturbed metabolic network. Science.

[26] Chatr-Aryamontri A, Breitkreutz BJ, Oughtred R, Boucher L, Heinicke S, Chen D, Stark C, Breitkreutz A, Kolas N, O’Donnell L (2015). The biogrid interaction database: 2015 update. Nucleic Acids Res.

[27] Szklarczyk D, Franceschini A, Wyder S, Forslund K, Heller D, Huerta-Cepas J, Simonovic M, Roth A, Santos A, Tsafou KP (2015). String v10: protein–protein interaction networks, integrated over the tree of life. Nucleic Acids Res.

[28] Tuomela S, Salo V, Tripathi SK, Chen Z, Laurila K, Gupta B, Äijö T, Oikari L, Stockinger B, Lähdesmäki H (2012). Identification of early gene expression changes during human th17 cell differentiation. Blood.

[29] Smyth GK (2005). Limma: linear models for microarray data. Bioinformatics and Computational Biology Solutions Using R and Bioconductor.

[30] Flicek P, Amode MR, Barrell D, Beal K, Billis K, Brent S, Carvalho-Silva D, Clapham P, Coates G, Fitzgerald S (2014). Ensembl 2014. Nucleic Acids Res.

[31] Subramanian A, Tamayo P, Mootha VK, Mukherjee S, Ebert BL, Gillette MA, Paulovich A, Pomeroy SL, Golub TR, Lander ES (2005). Gene set enrichment analysis: a knowledge-based approach for interpreting genome-wide expression profiles. Proc Natl Acad Sci.

[32] Warde-Farley D, Donaldson SL, Comes O, Zuberi K, Badrawi R, Chao P, Franz M, Grouios C, Kazi F, Lopes CT (2010). The genemania prediction server: biological network integration for gene prioritization and predicting gene function. Nucleic Acids Res.

[33] Brummelman J, Raeven RH, Helm K, Pennings JL, Metz B, van Eden W, van Els CA, Han WG (2016). Transcriptome signature for dampened th2 dominance in acellular pertussis vaccine-induced cd4+ t cell responses through tlr4 ligation. Scientific reports.

[34] Suddason T, Gallagher E (2016). Genetic insights into map3k-dependent proliferative expansion of t cells. Cell Cycle.

[35] Cleret-Buhot A, Zhang Y, Planas D, Goulet JP, Monteiro P, Gosselin A, Wacleche VS, Tremblay CL, Jenabian MA, Routy JP (2015). Identification of novel hiv-1 dependency factors in primary ccr4+ ccr6+ th17 cells via a genome-wide transcriptional approach. Retrovirology.

[36] Wei L, Laurence A, Elias KM, O’Shea JJ (2007). Il-21 is produced by th17 cells and drives il-17 production in a stat3-dependent manner. J Biol Chem.

